# An Improved DOA Estimation Approach Using Coarray Interpolation and Matrix Denoising

**DOI:** 10.3390/s17051140

**Published:** 2017-05-16

**Authors:** Muran Guo, Tao Chen, Ben Wang

**Affiliations:** 1 College of Information and Communication Engineering, Harbin Engineering University, No. 145 Nantong Street, Harbin 150001, China; guomuran@hrbeu.edu.cn; 2 College of Automation, Harbin Engineering University, No. 145 Nantong Street, Harbin 150001, China; wangben@hrbeu.edu.cn; 3 Depaprtment of Electrical and Computer Engineering, Temple University, Philadelphia, PA 19122, USA

**Keywords:** array interpolation, direction-of-arrival estimation, matrix denoising, MUSIC, nuclear norm minimization

## Abstract

Co-prime arrays can estimate the directions of arrival (DOAs) of O(MN) sources with O(M+N) sensors, and are convenient to analyze due to their closed-form expression for the locations of virtual lags. However, the number of degrees of freedom is limited due to the existence of holes in difference coarrays if subspace-based algorithms such as the spatial smoothing multiple signal classification (MUSIC) algorithm are utilized. To address this issue, techniques such as positive definite Toeplitz completion and array interpolation have been proposed in the literature. Another factor that compromises the accuracy of DOA estimation is the limitation of the number of snapshots. Coarray-based processing is particularly sensitive to the discrepancy between the sample covariance matrix and the ideal covariance matrix due to the finite number of snapshots. In this paper, coarray interpolation based on matrix completion (MC) followed by a denoising operation is proposed to detect more sources with a higher accuracy. The effectiveness of the proposed method is based on the capability of MC to fill in holes in the virtual sensors and that of MC denoising operation to reduce the perturbation in the sample covariance matrix. The results of numerical simulations verify the superiority of the proposed approach.

## 1. Introduction

Array signal processing contains a wide area of research, such as array signal parameter estimation [1] and beamforming [2,3]. Direction-of-arrival (DOA) estimation is a vital problem in array signal parameter estimation [4,5,6,7,8,9], and is widely used in practical systems, such as telemedicine systems [10] and industrial high-voltage insulation systems [11]. One area in DOA estimation that has gained considerable interest is the estimation of the DOAs of as many signals as possible under a given number of sensors. Toward this purpose, many sparse linear array structures have been proposed to achieve a higher number of degrees of freedom (DOFs) than a uniform linear array (ULA) by exploiting the corresponding difference coarray. For instance, the minimum redundancy array (MRA) [12] can maximize the number of consecutive virtual sensors with a given number of physical sensors. However, there are no general expressions about how to determine the locations of physical sensors in an MRA, thus making it difficult for systematic design and optimizations.

Compared with the MRA, the nested array [13] and the co-prime array [14] are more attractive because of the systematic design of the physical sensors and the closed-form expressions of the corresponding virtual lags. A nested array can detect $O(N^{2})$ sources with O(N) physical sensors by simply combining two uniform linear subarrays. Different from an MRA, a nested array has a fixed structure which is convenient to analyze. Another advantage of this structure is that the corresponding difference virtual lags are all contiguous. This is very friendly for the implementation of the spatial smoothing-based multiple signal classification (SS-MUSIC) method [1,15]. However, the nested array requires one subarray with half waveform interelement spacing, which will cause the mutual coupling effect [16,17]. Comparatively, co-prime arrays consist of two subarrays with a co-prime number of physical sensors, and can achieve a higher number of DOFs with negligible mutual coupling effects. The performance of a co-prime array is analyzed explicitly in [14].

MUSIC is a commonly-used high-resolution DOA estimation technique that exploits the orthogonality between the signal subspace and the noise subspace. Because the covariance matrix obtained from coarray lags is rank one, and thus spatial smoothing (SS) has to be applied to restore the full matrix rank, only contiguous virtual lags can be used if spatial smoothing MUSIC (SS-MUSIC) is applied to estimate DOAs under a co-prime array configuration. If the holes can be filled by the data of other lags, then the lags beyond contiguous range can also be utilized to recognize more sources. Matrix completion is a recently developed technique which is essentially an extension of compressive sensing [18,19] aiming to recover low rank matrices with missing samples. Theoretically, if a matrix with dimension $n_{1}\times n_{2}$ has a low rank, then it can be reconstructed from O(nrpolylog(n)) entries, where $n=\max\{n_{1},n_{2}\}$ and *r* is the rank of the corresponding matrix [19]. For DOA estimation, the rank of the covariance matrix of a coarray equals to the number of sources, and it is smaller than the number of unique virtual lags, which guarantees that the covariance matrix is low rank. Thus, the holes in coarray can be filled with matrix completion [20]. Various algorithms have been proposed to solve the matrix completion problem, such as singular value thresholding (SVT) [21] and fixed point continuation (FPC) [22].

In practice, the covariance matrix of a received signal is estimated using finite snapshots; i.e., ${\tilde{R}}_{S}=\sum_{k=1}^{K}{\tilde{x}}_{S}\left[k\right]{\tilde{x}}_{S}^{H}\left[k\right]$. In this case, an estimation error will occur—particularly when the number of snapshots is small. To solve this problem, an approach has been proposed in [23] for nested arrays by exploiting the nuclear norm minimization problem. Since the nested array forms a hole-free coarray, the same method cannot be readily applied for the co-prime array that has holes in the corresponding coarray. In addition, the optimal result of the nuclear norm minimization problem in [23] is not necessarily Hermitian, so the MUSIC method has to be modified for spatial spectrum estimation based on singular value decompositions.

In this paper, a novel approach is proposed to achieve accurate DOA estimation with a high number of DOFs. Coarray interpolation is first performed in the proposed approach to fill the holes and thus achieve a high number of consecutive coarray elements. Then, an improved denoising operation which reconstructs the covariance matrix by exploiting the Hermitian Toeplitz structure is employed to suppress the errors in the data sample covariance matrix due to the finite number of snapshots. Finally, the MUSIC technique is used to estimate the DOAs without spatial smoothing operation. The main contribution of this paper is the improved denoising operation, in which the Hermit and Toeplitz property of the covariance matrix are exploited. As such, the complexity of the denoising operation is significantly reduced. Furthermore, a subspace-based algorithm such as MUSIC can be directly used to estimate the DOAs. Numerical experiments are conducted to verify the effectiveness of the proposed approach.

The rest of this paper is organized as follows. In Section 2, we set up the system model used through this paper. Then, the methods of the MUSIC algorithm and coarray interpolation are elaborated in Section 3. The theory of the new DOA estimate approach and the corresponding process are given in Section 4. The results of numerical experiments are shown in Section 5. In Section 6, a summary of this paper is provided.

Notations: Scalars, vectors, matrices, and sets are denoted by lower-case letters (a), lower-case bold letters (a), upper-case bold letters (A), and upper-case letters in blackboard boldface (A), respectively. Integer set, real number set, and complex number set are respectively denoted by Z, R, and C. Especially, $I_{M}$ denotes the identity matrix with dimension M×M. $A^{*}$, $A^{T}$, and $A^{H}$ denote the conjugate, the transpose, and the complex conjugate transpose of A. ⊗ denotes the Kronecker product. vec(·) denotes the vectorization operator that turns a matrix into a vector by stacking all columns on top of the another, and diag(a) denotes a diagonal matrix that uses the elements of a as its diagonal elements. |A| is the cardinality of A. tr(A) represents the trace of matrix A. ${∥A∥}_{*}$ and ${∥A∥}_{F}$, respectively, denote the nuclear norm and the Frobenius norm of matrix A. E[·] denotes the expectation operator. ${\left[A\right]}_{i,j}$ indicates the (i,j)th entry of A. The square bracket notation of a vector ${\left[x_{S}\right]}_{i}$ represents the *i*th component of $x_{S}$. The triangular bracket notation ${\langle x_{S}\rangle }_{n}$ denotes the signal value on the support n∈S, and is very useful for nonuniform arrays. For instance, if S={1,3,5,7} and $x_{S}={\left[1,2,3,4\right]}^{T}$, then the square brackets ${\left[x_{S}\right]}_{1}=1$, ${\left[x_{S}\right]}_{2}=2$,${\left[x_{S}\right]}_{3}=3$ and ${\left[x_{S}\right]}_{4}=4$, and the triangular brackets ${\langle x_{S}\rangle }_{1}=1$, ${\langle x_{S}\rangle }_{3}=2$, ${\langle x_{S}\rangle }_{5}=3$ and ${\langle x_{S}\rangle }_{7}=4$.

## 2. System Model

An extended co-prime array which is generated by the co-prime integers pair (M,N) was proposed in [24]. Without loss of generality, assume that M<N. Then, the extended co-prime array consists of two subarrays: one having 2M sensors with an inter-element spacing of *N* units, and the other having *N* sensors with an inter-element spacing of *M* units. To be precise, the *L* physical sensors have locations $L=\left\{p_{1},p_{2},\cdots ,p_{L}\right\}=S\times d_{0}$, where S is an integer set given by

(1) S={Mn|0≤n≤N−1}∪{Nm|0≤m≤2M−1},

and $d_{0}=\lambda /2$ is the unit inter-element spacing, with λ denoting the wavelength. Then, the locations of the corresponding difference coarray are denoted as $D\times d_{0}$, where D is given by

(2) $$D=\{c_{1}-c_{2}|c_{1},c_{2}\in S\}.$$

An example of the extended co-prime array is given in Example 1.

**Figure 1:**
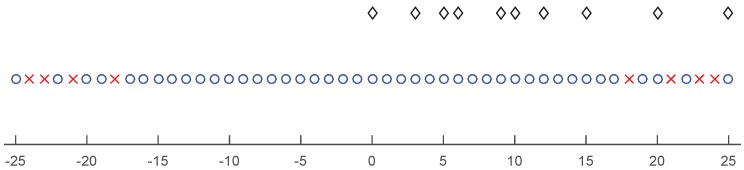
An example extended co-prime array configuration with M=3 and N=5. The black diamonds are physical sensors. The blue circle represents the difference coarray, while the red cross represents the holes.

**Example** **1.** Let M=3 and N=5. The corresponding integer set of the physical sensor positions is S={0,3,5,6,9,10,12,15,20,25}. The corresponding difference coarray is D={0,±1,⋯,±17,±19,±20,±22,±25}. The array configuration of this example is shown in Figure 1.

Denote $s\left(t\right)={\left[s_{1}\left(t\right),\cdots ,s_{Q}\left(t\right)\right]}^{T}$ as the source signal vector which follows the unconditional model (stochastic model) [25]. Under the unconditional model, the source signal vector s(t) is assumed to be a Gaussian random vector with mean zero and covariance $P=E\left[s\left(t\right)s^{H}\left(t\right)\right]$. n(t) is the additive white Gaussian noise vector, which is uncorrelated to the source signals. *Q* is the number of sources, and is assumed to be known throughout this paper. In addition, we assume that the sources are uncorrelated. Then, the baseband received signal vector at time index *t* can be expressed as

(3) $$x_{S}\left(t\right)=A_{S}s\left(t\right)+n\left(t\right),$$

where $A_{S}=\left[a_{S}\left(\theta_{1}\right),\cdots ,a_{S}\left(\theta_{Q}\right)\right]$ is the manifold matrix and $a_{S}\left(\theta_{q}\right)$ is the steering vector corresponding to the *q*th source, expressed by

(4) $$a_{S}\left(\theta_{q}\right)={\left[e^{\frac{j2\pi p_{1}d_{0}\sin\left(\theta_{q}\right)}{\lambda }},\cdots ,e^{\frac{j2\pi p_{L}d_{0}\sin\left(\theta_{q}\right)}{\lambda }}\right]}^{T},\;q=1,2,\cdots ,Q.$$

Let $R_{S}=E\left[x_{S}\left(t\right)x_{S}^{H}\left(t\right)\right]$ denote the covariance matrix of $x_{S}\left(t\right)$. According to (3) , $R_{S}$ can be written as

(5) $$R_{S}=\sum_{q=1}^{Q}\sigma_{q}^{2}a_{S}\left(\theta_{q}\right)a_{S}^{H}\left(\theta_{q}\right)+\sigma_{n}^{2}I_{L}=A_{S}R_{S}A_{S}^{H}+\sigma_{n}^{2}I_{L},$$

where $R_{S}=\mathrm{diag}\left(\sigma_{1}^{2},\cdots ,\sigma_{Q}^{2}\right)$ is the covariance matrix of source signals. $\sigma_{q}^{2}$ denotes the power of the *q*th source, while $\sigma_{n}^{2}$ is the variance of the noise. In practice, only a finite number of snapshots of the received signal are available to estimate the covariance matrix. Let $x_{S}\left[k\right]$ with k=1,2,⋯,K be the *K* snapshots of the received signal. Then, the maximum likelihood (ML) estimation of $R_{S}$—denoted by ${\tilde{R}}_{S}$—is

(6) $${\tilde{R}}_{S}=\sum_{k=1}^{K}x_{S}\left[k\right]x_{S}^{H}\left[k\right].$$

Throughout this paper, we use $\tilde{\cdot }$ to represent the estimation of the corresponding vector or matrix, unless it is otherwise specified.

The autocorrelations of sensor output signal evaluated at lag set D—denoted by $x_{D}\in C^{|D|\times 1}$—can be derived by reshaping $R_{S}$ as follows

(7) $$x_{D}=F\mathrm{vec}\left(R_{S}\right)=A_{D}b+\sigma_{n}^{2}e_{0},$$

where $A_{D}$ is the manifold matrix of the coarray, $b={\left[\sigma_{1}^{2},\cdots ,\sigma_{Q}^{2}\right]}^{T}$ is the power of sources, and $e_{0}$ is a column vector satisfying ${\langle e_{0}\rangle }_{m}=\delta_{m,0}$. F is *the transform matrix* of dimension $\left|D\right|\times {\left|S\right|}^{2}$, which is defined by the following definitions:

**Definition** **1.** The array weight function [13] ω(l):Z↦Z is defined by $\omega \left(l\right)=|\{\left(c_{1},c_{2}\right)\in S^{2}|c_{1}-c_{2}=l\}|$.

**Definition** **2.** *The transform matrix [26] F is a real matrix of size $\left|D\right|\times {\left|S\right|}^{2}$ such that the row of F associated with the difference $m_{0}\in D$ is defined by*

$${\langle F\rangle }_{m_{0},:}=\mathrm{vec}{\left(I\left(m_{0}\right)\right)}^{T},$$

*where the |S|×|S| matrix $I(m_{0})$ satisfies*

(8) $${\langle I\left(m_{0}\right)\rangle }_{c_{1},c_{2}}=\left\{\begin{array}{cc} \frac{1}{\omega (m_{0})}, & if\;c_{1}-c_{2}=m_{0},\\ 0, & otherwise. \end{array}\right.\;c_{1},c_{2}\in S.$$

*Vectorizing $R_{S}$ yields*

(9) $$\begin{array}{cc} \mathrm{vec}(R_{S}) & =\sum_{q=1}^{Q}\sigma_{q}^{2}\mathrm{vec}\left(a_{S}\left(\theta_{q}\right)a_{S}^{H}\left(\theta_{q}\right)\right)+\sigma_{n}^{2}\mathrm{vec}\left(I\right)\\ & =\sum_{q=1}^{Q}\sigma_{q}^{2}a_{S}^{*}\left(\theta_{q}\right)⊗a_{S}\left(\theta_{q}\right)+\sigma_{n}^{2}\mathrm{vec}\left(I\right). \end{array}$$

## 3. Coarray Interpolation

### 3.1. MUSIC Algorithm Based on Coarray

We can find that (7) has a similar form with (3). As such, $x_{D}$ can be regarded as a *deterministic* data vector on the difference coarray D. However, only one snapshot is available. As such, this problem is similar to handling fully coherent sources. The subspace-based DOA estimation techniques (e.g., MUSIC) failed to yield reliable DOA estimates when multiple signals impinge on the array. To overcome this problem, a spatial smoothing technique [27] and a more direct approach [15] have been proposed. However, these two approaches require overlapping subarrays with the same structure. As such, only the contiguous lags can be used, which will reduce the number of DOFs. For simplicity, we only give the principle of SS-MUSIC next. The explicit elaboration of the direct augmentation-based MUSIC (DA-MUSIC) can be found in [15].

Denote $U=\{-l_{\xi },-l_{\xi }+1,\cdots ,l_{\xi }\}$ as the maximum central contiguous ULA segment and $x_{U}$ as the data vector on U. Then, we divide the virtual ULA into $l_{\xi }+1$ overlapping subarrays $\left\{x_{U1},\cdots ,x_{U(l_{\xi }+1)}\right\}$, where $x_{Ui}$ contains sensors located at −i+1+k with $i=1,2,\cdots ,l_{\xi }+1$ and $k=0,1,\cdots ,l_{\xi }$. Define $R_{i}=x_{Ui}x_{Ui}^{H}$, and take the average of $R_{i}$ over all *i*. Then, we obtain

(10) $$R_{ss}=\frac{1}{l_{\xi }+1}\sum_{i=1}^{l_{\xi }+1}R_{i}.$$

As such, the rank of $R_{ss}$ is recovered, and the MUSIC technique can be performed to estimate the DOAs. To obtain the signal and noise subspaces, the eigendecomposition of the covariance matrix $R_{ss}$ is expressed as

(11) $$R_{ss}=\left[\begin{array}{cc} U_{S} & U_{N} \end{array}\right]\Sigma \left[\begin{array}{c} U_{S}^{H}\\ U_{N}^{H} \end{array}\right],$$

where Σ is a diagonal matrix with elements of eigenvalues in descending order. $U_{S}\in C^{(l_{\xi }+1)\times Q}$ is the signal subspace consisting of *Q* eigenvectors corresponding to the *Q* largest eigenvalues, while $U_{N}\in C^{\left(l_{\xi }+1\right)\times \left(l_{\xi }-Q+1\right)}$ is the noise subspace consisting of $l_{\xi }-Q+1$ eigenvectors corresponding to the $l_{\xi }-Q+1$ smallest eigenvalues.

Based on the MUSIC technique, the signal subspace is orthogonal to the noise subspace. Note that the signal subspace is spanned by the steering vectors, which means that the steering vectors of sources are also orthogonal to the noise subspace; i.e.,

(12) $$a^{H}\left(\theta_{q}\right)U_{N}=0.$$

As such, the DOAs can be estimated by finding the peaks of the following spatial pseudo- spectrum function

(13) $$\hat{\theta }=\underset{\theta \in \vartheta }{\mathrm{argmax}}\frac{1}{a^{H}\left(\theta \right)U_{N}U_{N}^{H}a\left(\theta \right)},$$

where ϑ is the searching grid.

### 3.2. Co-Prime Coarray Interpolation

For co-prime array, the number of DOFs is limited if the MUSIC technique is applied to estimate the DOAs due to the existence of holes in coarray. However, if the holes can be interpolated, the remaining virtual lags beyond the contiguous range can be utilized to construct a ULA with a larger aperture. Define V as the shortest ULA containing D (namely, V={m|min(D)≤m≤max(D)}), and the corresponding covariance matrix of V can also be expressed using $x_{D}$ with missing entries. In detail, since the covariance matrix of a ULA is a Toeplitz matrix, we can set the data at non-positive support of $x_{D}$ as the first column of $R_{V}$ with missing samples on holes. Similarly, set the data at non-negative support of $x_{D}$ as the first row of $R_{V}$ with missing samples on holes. Then, we can get a Toeplitz matrix with missing entries, which is a basic form of MC [28]. The missing entries can be estimated by solving the nuclear norm minimization problem. The coarray interpolation method can be used to fill the holes based on MC [20] as follows:

(14) $$\begin{array}{cc} {\tilde{R}}_{V}^{☆}= & \underset{{\tilde{R}}_{V}\in C^{|V^{+}\left|\times \right|V^{+}|}}{\mathrm{argmin}}{∥{\tilde{R}}_{V}∥}_{*}\\ s.t.\; & {\tilde{R}}_{V}={\tilde{R}}_{V}^{H},\\ & {\langle {\tilde{R}}_{V}\rangle }_{n_{1},n_{2}}={\langle {\tilde{x}}_{D}\rangle }_{n_{1}-n_{2}}, \end{array}$$

where $V^{+}=\left\{n|n\in V,n\geq 0\right\}$ is the non-negative part of V. The range of $n_{1}$ and $n_{2}$ is $\left\{n_{1},n_{2}|n_{1},n_{2}\in V^{+},n_{1}-n_{2}\in D\right\}$. By solving (14), all the known correlation information of ${\tilde{x}}_{D}$ is contained in ${\tilde{R}}_{V}$. The optimal solution of (14), ${\tilde{R}}_{V}^{☆}$, can be directly utilized to compute the coarray MUSIC spectrum. However, the DOFs are still limited by the cardinality of D, which is smaller than that of V.

## 4. Hybrid Approach

As mentioned in Section 2, the covariance matrix ${\tilde{R}}_{S}$ is calculated for DOA estimation with a finite number of snapshots. As such, there exists an error ${\tilde{E}}_{0}$ due to the finite snapshots, which is the common case. Then, the ${\tilde{R}}_{S}$ can be rewritten as

(15) $${\tilde{R}}_{S}=A_{S}R_{S}A_{S}^{H}+\tilde{E}.$$

$\tilde{E}$ contains two components—i.e., the power matrix of noise $\sigma_{n}^{2}I$ and error caused by finite snapshots ${\tilde{E}}_{0}$. After a series of operations (e.g., reshaping and coarray interpolation), $\tilde{E}$ still exists in another form, denoted as ${\tilde{E}}_{1}$. Then, we can rewrite ${\tilde{R}}_{V}$ as

(16) $${\tilde{R}}_{V}=R_{V}+{\tilde{E}}_{1}.$$

A denoising method has been proposed to eliminate this kind of error in spatial smoothing covariance matrix [23]. However, in [23], the structure of the covariance matrix is not exploited, so the optimal solution may not be Hermitian. As such, only a MUSIC-like spectrum defined by singular value decomposition can be used to estimate DOAs. Inspired by this denoising method and coarray interpolation, we propose an improved DOA estimation approach to achieve a better estimation accuracy and simultaneously guarantee high DOFs.

In this approach, a coarray interpolation method is used to get ${\tilde{R}}_{V}$ from (14), which acquires higher DOFs than the spatial smoothing technique at first. Then, a denoising operation follows to suppress the error ${\tilde{E}}_{1}$. It is well known that the covariance matrix of ULA is a Toeplitz and Hermitian matrix. To be precise, $R_{V}$ has the following form:

(17) $$R_{V}=T\left(u\right)=\left[\begin{array}{cccc} u_{1} & u_{2} & \cdots & u_{|V^{+}|}\\ u_{2}^{H} & u_{1} & \cdots & u_{|V^{+}|-1}\\ \vdots & \vdots & \ddots & \vdots \\ u_{|V^{+}|} & u_{|V^{+}|-1} & \cdots & u_{1} \end{array}\right],$$

where $u=\left[u_{1},\cdots ,u_{|V^{+}|}\right]$ is the first row of $R_{V}$. As such, a denoising measure is designed to eliminate error by optimizing the following nuclear norm minimization problem

(18) $$\begin{array}{cc} & \min_{u}{∥T\left(u\right)∥}_{*}\\ s.t.\; & ∥{\tilde{R}}_{V}{-T\left(u\right)∥}_{F}\leq \epsilon , \end{array}$$

where the ϵ is related to the noise level. Equivalently, (18) can be reformulated in the regularization form

(19) $$\begin{array}{c} \min_{u}{\mu ∥T\left(u\right)∥}_{*}+\frac{1}{2}{∥{\tilde{R}}_{V}-T\left(u\right)∥}_{F}, \end{array}$$

where μ is the regularization parameter.

In (14), the DOF of ${\tilde{R}}_{V}$ is $O(|V^{+}{|}^{2})$. However, by exploiting the Toeplitz and Hermitian property of $R_{V}$, vector u contains all of the unique elements of $R_{V}$, and the corresponding DOF is reduced to $O(|V^{+}|)$ in (18). Thus, the computational complexity decreases significantly by optimizing u instead of the entire $R_{V}$. The advantages of the proposed approach are summarized below:

1. The co-prime coarray interpolation step is used to fill the holes. As a result, the lags out of the contiguous range are utilized, leading to a higher number of DOFs than the SS-MUSIC, which only uses the contiguous lags.
2. The full rank covariance matrix can be readily established by optimizing (14) from $x_{D}$ without a spatial smoothing step. This operation can reduce the complexity and is easy to perform.
3. In (18), the structure of the covariance matrix of ULA is fully exploited. Thus, the optimal covariance matrix acquired by u is Toeplitz and Hermitian, thus enabling effective DOA estimation using a subspace-based algorithm such as the MUSIC. In addition, the complexity is reduced by fully exploiting the structure of $R_{V}$. Furthermore, the error matrix ${\tilde{E}}_{1}$ is also suppressed effectively, which is the main purpose of the denoising step.

Due to the above advantages of the proposed approach, a more accurate estimation of the covariance matrix is achieved, while a higher number of resolvable sources is guaranteed.

Next, we can estimate DOAs using the MUSIC defined in (13), where the noise subspace $U_{N}$ is replaced by $U_{N_{V}}$, obtained from $R_{V}^{☆}$. In addition, we use the sensor array located at $V\times d_{0}$ as the virtual array and

(20) $$a_{V}\left(\theta \right)=\left[1,e^{\frac{j2\pi d_{0}\sin\left(\theta \right)}{\lambda }},\cdots ,e^{\frac{j2\pi \max\left(V\right)d_{0}\sin\left(\theta \right)}{\lambda }}\right]$$

as the corresponding steering vector. Substituting $a_{V}\left(\theta \right)$ and $U_{N_{V}}$ into (13), the DOAs can be estimated by finding the *Q* largest peaks of the MUSIC pseudo-spectrum function. The entire approach is tabulated in Table 1.

## 5. Simulation Results

In this section, a series of simulations are conducted to examine the performance of the proposed approach. We first compare the spatial spectrum between the interpolated coarray and the proposed approach. Then, Monte Carlo experiments are conducted to examine the performance versus the input signal-to-noise ratio (SNR) and the number of snapshots. Angular resolution is examined in the third subsection. Note that MUSIC is used to estimate the DOAs by searching from $-90^{\circ }$ to $90^{\circ }$ with step $0.1^{\circ }$.

In the following simulations, the signal power of sources is assumed to be the same. In addition, SNR is defined as

$$\mathrm{SNR}=10\log\frac{\sigma^{2}}{\sigma_{n}^{2}}=20\log\frac{\sigma }{\sigma_{n}},$$

where $\sigma^{2}$ is the signal power of sources, while $\sigma_{n}^{2}$ is the variance of noise.

To evaluate the performance of the proposed method, the average root mean square error (RMSE) of the estimated DOAs is defined as

$$\mathrm{RMSE}=\sqrt{\frac{1}{IQ}\sum_{i=1}^{I}\sum_{q=1}^{Q}{\left({\tilde{\theta }}_{q}\left(i\right)-\theta_{q}\right)}^{2}},$$

where ${\tilde{\theta }}_{q}\left(i\right)$ is the estimate of $\theta_{q}$ for the *i*th Monte Carlo experiment, i=1,2,⋯,I. We conduct I=500 independent experiments in the peformance analysis subsection.

### 5.1. MUSIC Spectrum Analysis

**Figure 2:**
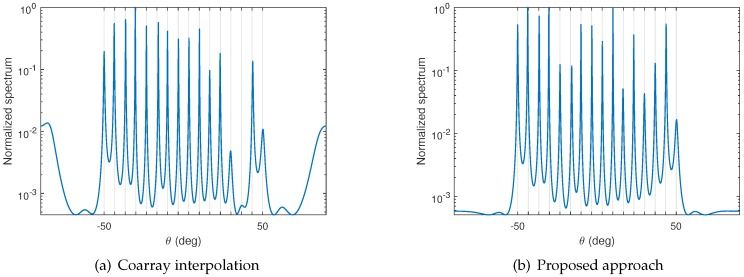
Spectrum comparison between two approaches. The number of snapshots is K=100 and signal-to-noise ratio (SNR) is 0dB. (**a**) Coarray interpolation in [20]; (**b**) Proposed approach.

In this subsection, we compare the MUSIC spectrum obtained from the coarray interpolation approach and the proposed approach. A co-prime array consisting of 10 sensors with location S={0,3,5,6,9,10,12,15,20,25} is considered throughout this subsection. The corresponding difference set is D={0,±1,⋯,±17,±19,±20,±22,±25}, and the maximum contiguous set obtained after interpolation is V={0,±1,⋯,±25}. In addition, Q=16 far-field narrowband sources distributed uniformly from $-50^{\circ }$ to $50^{\circ }$ are assumed to impinge on the array. SVT is used to obtain the optimal solution of (14). The regularization parameter in (19) is empirically selected as μ=0.1. In this scenario, the SNR is 0dB, and the number of snapshots is K=100. The MUSIC spectra are compared in Figure 2. For the coarray interpolation case depicted in Figure 2a, the amplitudes of spurious peaks are higher than the true peaks due to the low number of snapshots and SNR. This will lead to false DOA estimation. For the hybrid approach shown in Figure 2b, the spurious peaks are successfully suppressed due to the denoising operation. As such, precise DOA estimation is obtained.

### 5.2. Estimation Performance Analysis

**Figure 3:**
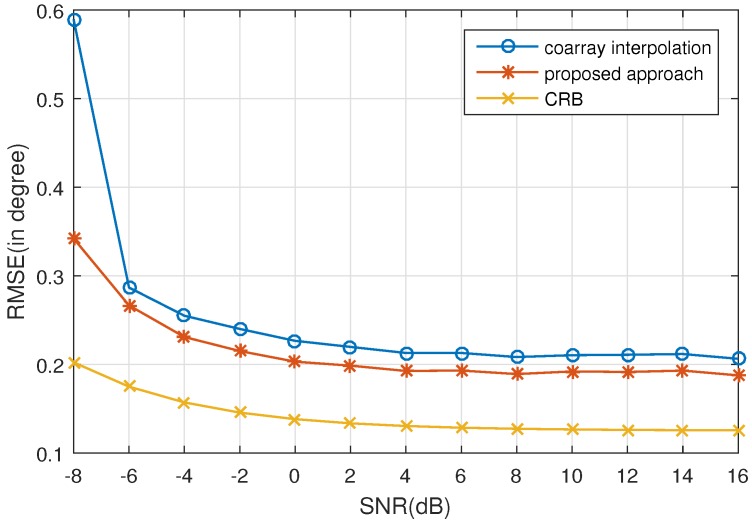
Root mean square error (RMSE) vs. SNR for 500 Monte Carlo experiments with *Q* = 16 sources uniformly distributed in $\left[-50^{\circ },50^{\circ }\right]$. The number of snapshots is K=500. CRB: Cramér–Rao Bound.

**Figure 4:**
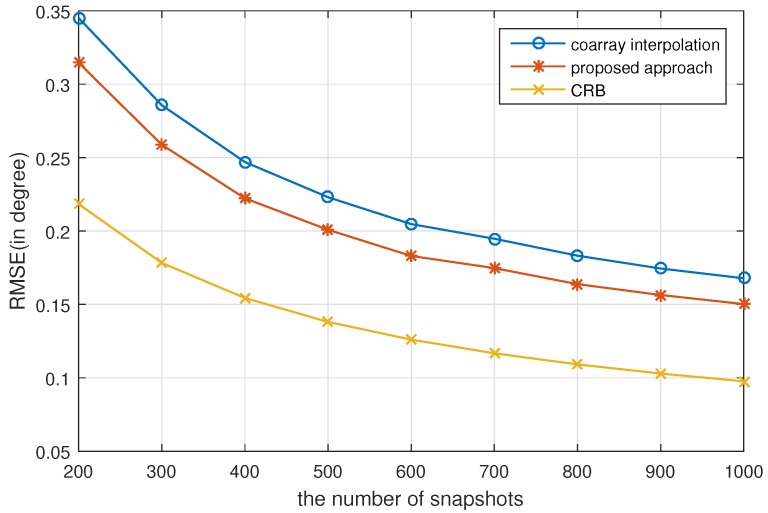
RMSE vs. the number of snapshots for 500 Monte Carlo experiments with Q=16 sources uniformly distributed in $\left[-50^{\circ },50^{\circ }\right]$. The SNR is 0dB.

In this subsection, the RMSE performance versus SNR and the number of snapshots is examined by conducting 500 Monte Carlo experiments. The simulation parameters are set the same as in Section 5.1. Figure 3 shows the RMSE versus SNR where the number of snapshots is set as K=500. Figure 4 shows the RMSE versus the number of snapshots, where the SNR is 0dB. It is obvious that the proposed approach outperforms coarray interpolation. As analyzed in [26], when the number of sources is higher than that of physical array sensors, the average efficiency, which is defined as

$$\kappa =\frac{\mathrm{tr}({\mathrm{CRB}}_{\theta })}{Q\times {\mathrm{MSE}}_{an}},$$

cannot reach 1. ${\mathrm{MSE}}_{an}$ is the analytical mean square error (MSE) of coarray-based MUSIC. It indicates that the RMSE of MUSIC cannot reach the corresponding Cramér–Rao Bound (CRB), which is consistent with the simulation results. The CRB curves presented in Figure 3 and Figure 4 are drawn with the equations elaborated in [29].

### 5.3. Angular Resolution Analysis

In this subsection, the performance of angular resolution is examined. We assume that two sources from directions $\left\{-0.4^{\circ },0.6^{\circ }\right\}$ impinge on the sensors. The number of snapshots is K=200, and the SNR is 0dB. The regularization parameter is selected as μ=0.15.

**Figure 5:**
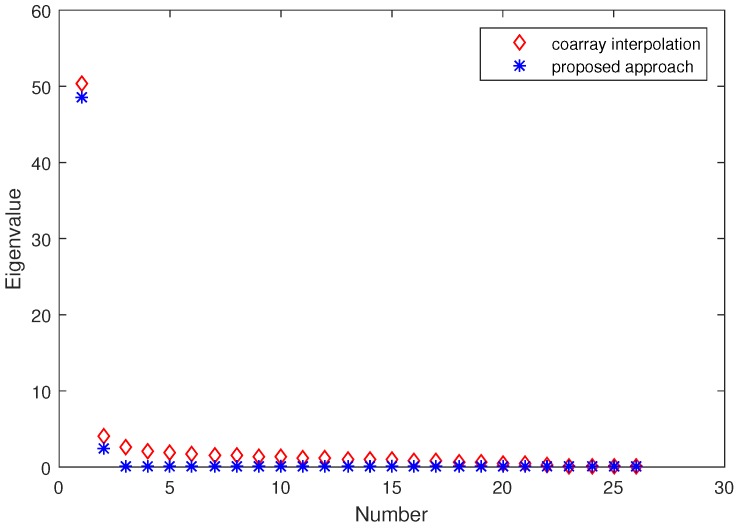
Sorted eigenvalues in the presence of two close sources. There are Q=2 sources located on $-0.4^{\circ }$ and $0.6^{\circ }$. The number of snapshots is K=200 and SNR is 0dB. The regularization parameter is set as μ=0.15.

In DOA estimation, the eigenvalue is a critical factor which affects the angular resolution. For this case or underlying two close sources, one of the signal eigenvalues is large while the other one is small. When the small signal eigenvalue is close to the largest noise eigenvalue, it will be difficult to distinguish the two sources. As such, to examine the angular resolution, we first examine the eigenvalues acquired from the coarray interpolation method and the proposed approach. The sorted eigenvalues are plotted in Figure 5 by averaging 100 Monte Carlo trials. It is clear that in the coarray interpolation method, the largest noise eigenvalue is comparable to the small signal eigenvalue. This will make the two close sources unrecognizable. For the proposed approach, the largest noise eigenvalue is nearly zero, and is much smaller than the small signal eigenvalue. In this case, the two close sources can be clearly distinguished.

**Figure 6:**
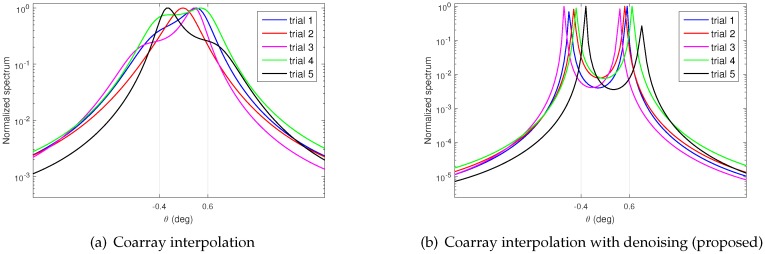
Spectrum for two close sources. There are Q=2 sources located on $-0.4^{\circ }$ and $0.6^{\circ }$. The number of snapshots is K=200, and SNR is 0dB. The regularization parameter is set as μ=0.15. (**a**) Use of only coarray interpolation [20]; (**b**) Use coarray interpolation followed by denoising (proposed).

In order to compare the angular resolution directly, Figure 6 gives the simulation results of five independent trials. In each trial, the same data vector is used to perform the two approaches. As shown in Figure 6a, we can see that the coarray interpolation cannot resolve the two close sources. As for the proposed approach, the two close sources are clearly resolved, as shown in Figure 6b. The errors are $0.3570^{\circ }$, $0.3002^{\circ }$, $0.4490^{\circ }$, $0.2364^{\circ }$, and $0.3669^{\circ }$, respectively. It is obvious that the angular resolution of the proposed approach is much better than the coarray interpolation.

## 6. Conclusions

An improved DOA estimation approach based on coarray interpolation and matrix denoising was proposed in this paper. The main contribution of this approach is the exploitation of both coarray interpolation and matrix denoising, where the former effectively increases the number of DOFs for coarray-based MUSIC algorithm, whereas the latter significantly reduces the estimation, please confirm that your intended meaning is retained error of the covariance matrix. In addition, the structure of the covariance matrix of ULA is fully exploited in the denoising operation, thus reducing the computational complexity. As a result, the DOA estimation accuracy and the angular resolution are significantly improved compared to the approach when only the coarray interpolation is applied. Simulation results verified the effectiveness of the proposed approach.

## Figures and Tables

**Table 1:** A novelty direction of arrival (DOA) estimation approach. MUSIC: multiple signal classification.

**Input**	The received signal vector ${\tilde{x}}_{S}\left[k\right]=A_{S}s\left[k\right]+n\left[k\right],k=1,2,\cdots ,K$.
**Output**	DOA Estimation.
**Step 1**	Compute the covariance matrix ${\tilde{R}}_{S}=\sum_{k=1}^{K}{\tilde{x}}_{S}\left[k\right]{\tilde{x}}_{S}^{H}\left[k\right]$.
**Step 2**	Reshape ${\tilde{R}}_{S}$ to get the signal vector of coarray $x_{D}=F\mathrm{vec}\left(R_{S}\right)$.
**Step 3**	Optimize (14) to get the covariance matrix ${\tilde{R}}_{V}$.
**Step 4**	Optimize (18) or (19) to get the denoised covariance matrix $R_{V}$.
**Step 5**	Perform eigenvalue decomposition of $R_{V}$ and construct $U_{N_{V}}=\left[u_{1},\cdots ,u_{|V^{+}|-Q}\right]$ where $\left\{u_{i},i=1,\cdots ,|V^{+}|-Q\right\}$ is the eigenvector corresponding to the $|V^{+}|-Q$ smallest eigenvalues.
**Step 6**	Compute $P_{MUSIC}=\frac{1}{a_{V}^{H}\left(\theta \right)U_{N_{V}}U_{N_{V}}^{H}a_{V}\left(\theta \right)}$ and find the *Q* largest peaks which correspond to the estimation of DOAs.
